# Barriers to Telemedicine Video Visits for Older Adults in Independent Living Facilities: Mixed Methods Cross-sectional Needs Assessment

**DOI:** 10.2196/34326

**Published:** 2022-04-19

**Authors:** Alice Mao, Lydia Tam, Audrey Xu, Kim Osborn, Meera Sheffrin, Christine Gould, Erika Schillinger, Marina Martin, Matthew Mesias

**Affiliations:** 1 Division of Primary Care and Population Health Department of Medicine Stanford University Stanford, CA United States; 2 On Lok Program of All-Inclusive Care for the Elderly San Jose, CA United States; 3 School of Medicine Stanford University Stanford, CA United States; 4 Stanford University Stanford, CA United States; 5 Geriatric Research Education and Clinical Center Veteran's Affairs Palo Alto Health Care System Palo Alto, CA United States; 6 Department of Psychiatry and Behavioral Sciences Stanford University Standford, CA United States

**Keywords:** telemedicine, barriers to access to care, older adults, eHealth, e-visit, access, accessibility, barrier, elder, gerontology, geriatric, need assessment, mixed method, cross-sectional, telehealth, community care, independent living

## Abstract

**Background:**

Despite the increasing availability of telemedicine video visits during the COVID-19 pandemic, older adults have greater challenges in getting care through telemedicine.

**Objective:**

We aim to better understand the barriers to telemedicine in community-dwelling older adults to improve the access to and experience of virtual visits.

**Methods:**

We conducted a mixed methods needs assessment of older adults at two independent living facilities (sites A and B) in Northern California between September 2020 and March 2021. Voluntary surveys were distributed. Semistructured interviews were then conducted with participants who provided contact information. Surveys ascertained participants’ preferred devices as well as comfort level, support, and top barriers regarding telephonic and video visits. Qualitative analysis of transcribed interviews identified key themes.

**Results:**

Survey respondents’ (N=249) average age was 84.6 (SD 6.6) years, and 76.7% (n=191) of the participants were female. At site A, 88.9% (111/125) had a bachelor’s degree or beyond, and 99.2% (124/125) listed English as their preferred language. At site B, 42.9% (51/119) had a bachelor’s degree or beyond, and 13.4% (16/119) preferred English, while 73.1% (87/119) preferred Mandarin. Regarding video visits, 36.5% (91/249) of all participants felt comfortable connecting with their health care team through video visits. Regarding top barriers, participants at site A reported not knowing how to connect to the platform (30/125, 24%), not being familiar with the technology (28/125, 22.4%), and having difficulty hearing (19/125, 15.2%), whereas for site B, the top barriers were not being able to speak English well (65/119, 54.6%), lack of familiarity with technology and the internet (44/119, 36.9%), and lack of interest in seeing providers outside of the clinic (42/119, 35.3%). Three key themes emerged from the follow-up interviews (n=15): (1) the perceived limitations of video visits, (2) the overwhelming process of learning the technology for telemedicine, and (3) the desire for in-person or on-demand help with telemedicine.

**Conclusions:**

Substantial barriers exist for older adults in connecting with their health care team through telemedicine, particularly through video visits. The largest barriers include difficulty with technology or using the video visit platform, hearing difficulty, language barriers, and lack of desire to see providers virtually. Efforts to improve telemedicine access for older adults should take into account patient perspectives.

## Introduction

Telemedicine, the practice of medicine using technology to deliver care at a distance, is an innovation with increasing uptake across the United States [1,2]. During the COVID-19 pandemic, the availability of telemedicine has skyrocketed due to waivers from the Centers for Medicare & Medicaid Services and other insurance providers, which have decreased restrictions on telemedicine use and increased payment parity compared with in-person visits [3,4]. Telemedicine visits enable patients to receive care remotely; they decrease the risk of infectious exposure for patients who are more vulnerable and increase the ease of access to care by decreasing cost, transportation challenges, and time spent going to see outpatient providers [5,6]. Among Medicare Advantage enrollees from January to June 2020, the weekly number of telemedicine visits increased 20-fold compared with prepandemic periods [7]. Many are hopeful for continued widespread use even beyond the pandemic [8].

Despite the increasing availability of telemedicine and its many advantages, older adults experience high barriers to access compared to younger adults [9,10]. A Pew Research report published in 2017 demonstrates that, while there is increasing internet and home broadband use among older adults, increasing age is still associated with a lack of confidence in using electronic devices [11]. It is estimated that 38% of US adults older than 65 years are not ready for video visits and that 72% of adults older than 85 years are not ready for video visits due to difficulties with hearing, vision, speaking, cognition, or difficulty with access or familiarity with internet-enabled devices [9]. One study of homebound older adults found that 82% of patients in one home-based primary care program required assistance from a caregiver to participate in virtual visits [12]. Providers were aware that barriers for these patients included cognitive or sensory impairment, but they were not knowledgeable about key access-related issues, such as their patients’ internet connectivity, ability to pay for cellular plans, or video-capable device access.

While much has been published on the feasibility of telemedicine in older adults domestically and abroad [13-18], there is limited information regarding the challenges of telemedicine from the perspective of patients. Existing work in the United States tends to focus on homogeneous English-speaking older adults [19-22]. To address this gap, we investigated the top barriers to telemedicine visits from the perspectives of older adults with differing socioeconomic backgrounds and primary spoken languages in two independent living facilities in Northern California. Our goal is to better inform proposed solutions to improve telemedicine access for diverse community-dwelling older adults.

## Methods

### Overview

We conducted a mixed methods needs assessment of two independent living facilities in Northern California as part of a quality improvement project to increase telemedicine access. Voluntary surveys (Multimedia Appendix 1) in English, Chinese, and Russian were distributed to older adults residing in the independent living facilities at both sites. Surveys were distributed by staff at each site as paper or electronic surveys to ensure accessibility. Site A houses residents who are mostly middle and upper-middle class native English speakers. Site B provides subsidized senior housing and serves a large group of non–English-speaking residents. These two sites were chosen to better understand the needs of older adults with differing socioeconomic and language backgrounds.

Surveys queried demographic information including gender, education level, preferred language, and residents’ previous experiences and preferences with technology or devices. While five-point Likert scales assessed comfort level, support, desire for, and barriers regarding telephone and video visits (Multimedia Appendix 1), responses were categorized as “agree” if participants selected “agree” or “strongly agree,” and “disagree” if they selected “disagree” or “strongly disagree.” Caregivers served as proxies for residents who could not physically respond. Surveys were translated from English to Russian and Chinese by independent researchers (authors LT and AX) to better serve residents in site B.

Follow-up semistructured phone interviews were conducted with surveyed participants who provided their contact information and were willing to speak to investigators to elaborate on perceived barriers (n=15; 8 participants from site A and 7 from site B). The questions asked during these interviews are reproduced in Multimedia Appendix 2. These interviews were deidentified and then translated and transcribed. Interview analysis followed the tenets of thematic analysis as described by Clarke and Braun [23], in which an inductive approach was taken and emerging concepts from the interviews were tagged as codes and then grouped into categories and ultimately themes. All interviews were independently read and coded with descriptive labels by three investigators (authors AM, AX, and M Mesias). Investigators met to discuss the coded transcripts halfway through reading all the interviews to resolve any coding discrepancy and, through consensus, finalize a set of codes used to code the rest of the transcripts as well as to recode prior transcripts. Final descriptive codes and representative quotes were then coalesced into broad categories and reviewed to identify emerging themes through an iterative process of discussion and collective consensus.

### Ethical Considerations

Given that this is a quality improvement project and not human participant research, this study received institutional review board (IRB) exemption from the Stanford University (IRB Protocol 58211).

## Results

### Demographics

Of the 700 surveys distributed, 249 surveys were completed (245 by patients, 4 by caregiver proxies). There were 125 participants from site A, 69.3% (n=87) of whom were female. Site B had 119 participants, with 84.9% (101/119) being female participants. There were 5 participants that did not designate a site on their survey and were excluded from site-specific analyses. At site A, the average age of participants was 85.5 (SD 6.6) years, while at site B, the average age was 83 (SD 6.6) years. When combined, the average age of all participants was 84.3 (SD 6.7) years. At site A, 88.9% (111/125) of participants had a bachelor’s degree or beyond, and 99.2% (124/125) listed English as their preferred language. At Site B, 42.9% (51/119) had a bachelor’s degree or beyond, and 13.4% (16/119) preferred English, while 73.1% (87/119) preferred Mandarin. Demographic information of survey participants is recapitulated in Table 1.

**Table 1:** Demographic information of survey participants. Participants from two independent living facilities were selected to ascertain the barriers that older adults experience in accessing telemedicine and to conduct a quality improvement project.

		Site A (n=125)	Site B (n=119)	Combined (N=249)
Age (years), mean (SD)		85.5 (6.6)	83.0 (6.6)	84.3 (6.7)
**Age groups (years), n (%)**				
	60-69	0 (0.0)	3 (2.5)	3 (1.2)
	70-79	23 (18.4)	31 (26.1)	57 (22.9)
	80-89	70 (56.0)	63 (52.9)	134 (53.8)
	90-99	29 (23.2)	20 (16.8)	49 (19.7)
	≥100	0 (0.0)	3 (2.5)	3 (1.2)
**Gender, n (%)**				
	Male	38 (30.4)	14 (11.8)	53 (21.3)
	Female	87 (69.3)	101 (84.9)	191 (76.7)
	Unspecified	0 (0.0)	4 (3.4)	5 (2.0)
**Race/ethnicity, n (%)**				
	White	116 (92.8)	22 (18.5)	138 (55.4)
	Non-White	9 (7.2)	97 (81.5)	111 (44.6)
**Preferred language, n (%)**				
	English	124 (99.2)	16 (13.4)	143 (57.4)
	Other	1 (0.8)	103 (86.6)	106 (42.6)
**Level of education, n (%)**				
	No bachelor’s degree	14 (11.2)	65 (54.6)	79 (31.7)
	Bachelor’s degree and beyond	111 (88.8)	51 (42.9)	166 (66.7)

### Survey Responses Regarding Use and Interest in Telemedicine

**Figure 1:**
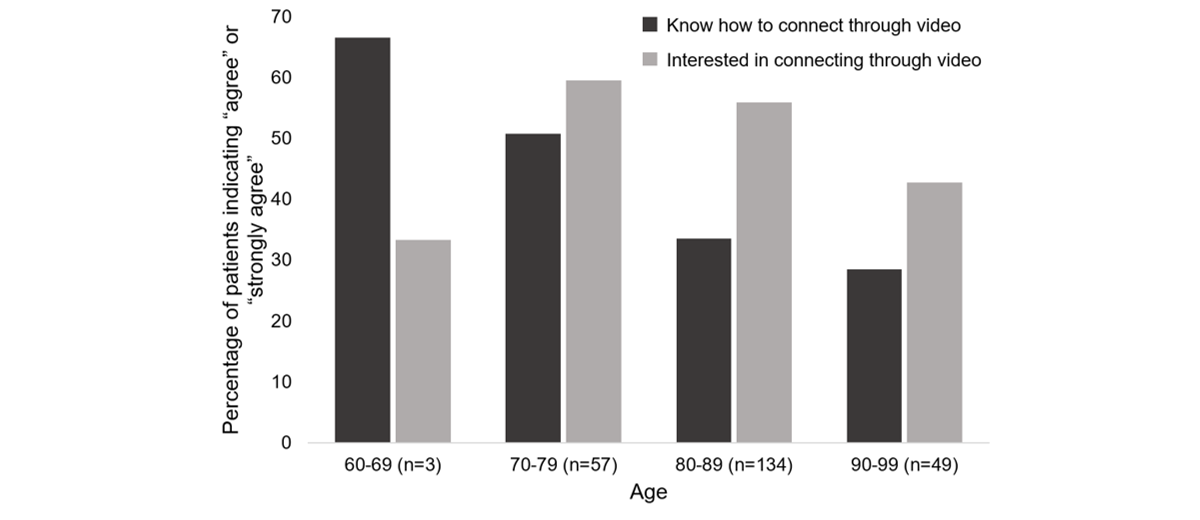
Age is associated with decreases in comfort with technology but not interest in telemedicine. While participants’ comfort with video visits decreased with increasing age (R2=0.96), interest in video visits was not associated with age (*R*^2^=0.07) in participants aged 60-99 years.

Regarding telemedicine visits, of the 249 participants 53% (n=132) of all participants were interested in connecting with their health care team through video visits, and 65.5% (n=163) preferred connecting through telephone. Regarding telemedicine comfort, 69.9% (174/249) of participants knew how to connect with their health care team through telephone. However, only 36.5% (n=91) knew how to connect with their health care team through video visits. Of those 91 participants, 68% (n=61) were from site A. For the 91 participants that were comfortable using video platforms, computers were the most preferred device (n=20, 23%), followed by smartphones (n=17, 19%) and iPads/tablets (n=10, 11%). We found that, while comfort with video visits decreased with increasing age (coefficient of determination, *R*^2^=0.96), it appears that increased age was not associated with decreased interest in telemedicine video visits (*R*^2^=0.07; Figure 1).

### Barriers Surrounding Telemedicine

**Figure 2:**
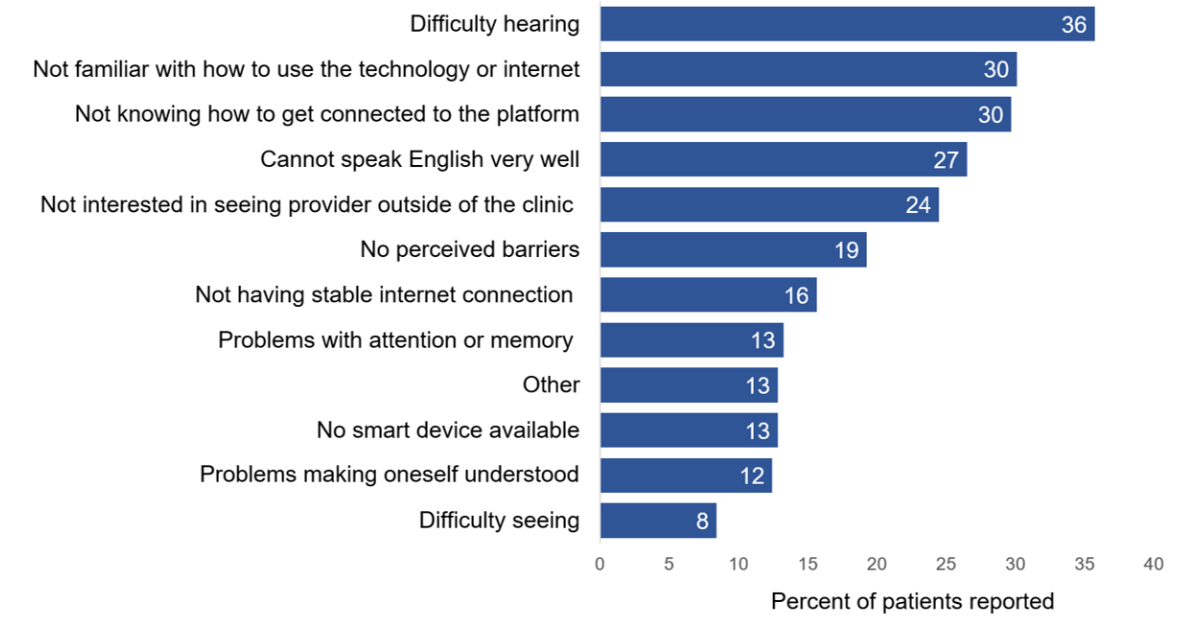
Perceived barriers to accessing telemedicine from both sites. Participants from both sites were asked to choose the biggest barriers (up to three) to connect with health care providers through video visits. Top perceived barriers reported include hearing difficulties, unfamiliarity with technology/internet or how to connect to the telemedicine platform, and language barriers.

The largest reported barriers to telemedicine visits for the 249 participants were hearing difficulties (n=89, 35.7%), not being familiar with how to use technology or the internet (n=75, 30.1%), not knowing how to get connected to the telemedicine platform (n=74, 29.7%), and language barriers (n=66, 26.5%; Figure 2). Of note, 65 of the 66 responses that indicated “cannot speak English very well” as a top barrier came from participants at site B. Other barriers from both sites included lack of interest in seeing providers outside of a clinic (n=61, 24.5%); poor internet connectivity (n=39, 15.7%) or lack of smart device (n=32, 12.9%); or difficulties with attention and memory (n=33, 13.3%), expressing oneself (n=31, 12.4%), or seeing (n=21, 8.4%).

**Figure 3:**
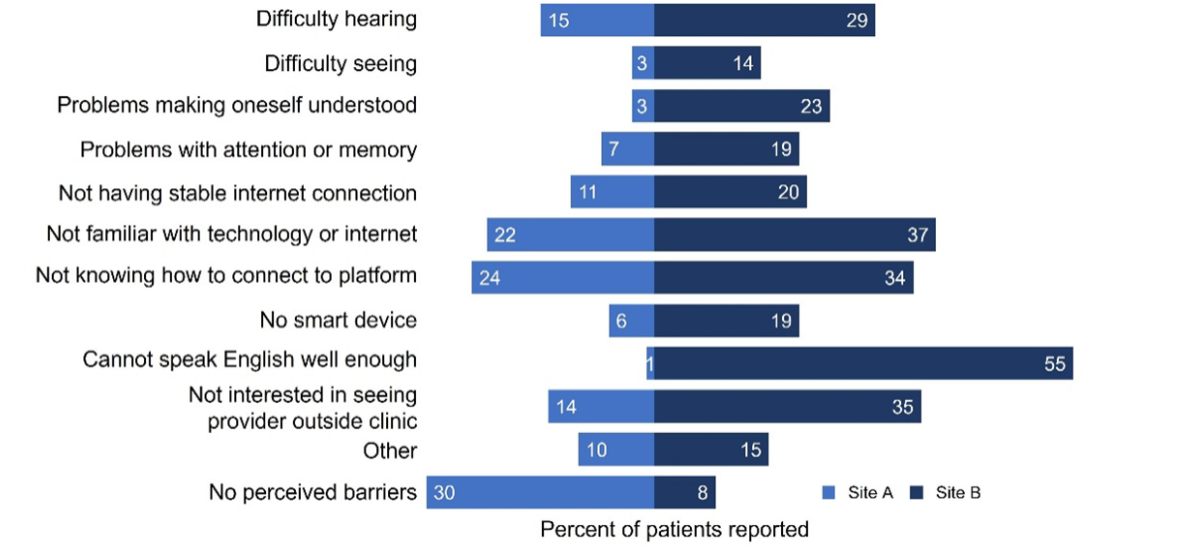
Site-specific perceived barriers to telemedicine access. When broken down by sites, top barriers differed. Site A participants cited unfamiliarity with technology/internet and connecting to the telemedicine platform, and hearing difficulties. Similarly, site B participants reported unfamiliarity with technology/internet and connecting to the telemedicine platform, though other major barriers included difficulty with English and lack of interest in seeing providers outside of the clinic.

Top barriers differed depending on the site (Figure 3). The top three barriers reported for the 125 participants at site A included not knowing how to connect to the platform (n=30, 24%), not being familiar with the technology (n=28, 22.4%), and difficulty hearing (n=19, 15.2%). At site A, 30% (n=37) of participants did not perceive any barriers to accessing telemedicine via video visits. The top barriers reported by 119 participants at site B included not being able to speak English well (n=65, 54.6%), not being familiar with the technology or internet (n=44, 37%), and lack of interest in seeing a provider outside of the clinic (n=42, 35.3%).

### Qualitative Themes

Several themes emerged from interviews exploring participants’ reported barriers regarding telemedicine perceived limitations of video visits, the overwhelming process of learning the technology for telemedicine, and desire for in-person or on-demand help with telemedicine.

#### Perceived Limitations of Video Visits

While most survey respondents expressed interest in connecting with their health care provider through telemedicine, many participants highlighted the limitations of telemedicine in comparison to in-person visits. One participant expressed the limitations of video visits in assuring a comprehensive workup:

Through video, there is no way to measure blood pressure...I can only tell you I don’t feel comfortable. If I were the doctor, hearing what I said, I would not know what to do because the symptoms are too broad.

Another participant noted that “video chats only solve part of the problem,” and that for regular checkups, she would still need to call and present herself to a health care facility. These perspectives highlight a perception of the lack of completeness of care when done through video visits.

Participants also described hesitancy to replace the much valued in-person experience with their health care provider. For instance, one participant stated:

I would rather that the doctor can actually touch me, examine me with a stethoscope, or see if a part is tender...I also think in-person communication is sometimes better...

Other participants describe preferring to speak to a human rather than technology and the experience of desiring personal contact and seeing expressions.

#### The Overwhelming Process of Learning the Technology for Telemedicine

One significant barrier surrounding telemedicine was the lack of familiarity with technology and the telemedicine platform. Some participants noted that they were intimidated by technology due to their age. One participant explained:

So I got an iPhone, it’s daunting as a 90-year-old. It’s got a billion buttons. I went out and purchased the manual, which is not produced by Apple--it’s produced by other people because Apple just presumes that people know how to use it [iPhone]

Some participants expressed familiarity with using technological devices for socialization and record keeping; the process of using unfamiliar with video platforms for telemedicine was more daunting. One participant highlighted this dichotomy well:

I have a really old device and I use it to keep in contact with relatives & keep updated with the news...There are many steps to book the [telemedicine] app, I have received a lot of information (eg. email) on how to connect. I feel like I am not smart enough to persist through the whole [set of instructions]

Others corroborated that they are reluctant to do video visits because “I just don’t enjoy setting it up.” They noted the process is cumbersome, and they need to experiment and get outside help before knowing how to navigate the platforms. Even for those who have successfully set up video visits in the past, the challenges of remembering how to log back on and remembering one’s password make the process difficult. One participant explained:

I can’t tell you how many times I’ve had to change my password...If my fingers hit the wrong button [the platform] notifies me the password is not working…We’ve had to reset the password 2-3 times and it took at least 2-3 hours to get a new password.

Some noted that they are familiar with platforms they already use such as Zoom or WeChat and would prefer if their care providers switched to simpler platforms for telemedicine video visits:

[The telemedicine platform is] very complicated -- much more so than Zoom. I have very poor vision and I’m old and it’s no good for me... I think just having help at the time I have to get on is the best or you should switch to a simpler system.

Furthermore, for the participants who did not speak English as their primary language, setting up telemedicine visits involved an added layer of difficulty as most of the instructions are in English. One participant elaborated:

If I use English, it will be very hard. I am comfortable with computers, and am willing to give it a try...I hope there is someone who speaks Chinese to help with technology.

Some participants reported relying on a spouse, an adult child caregiver, or a social worker to aid with these language barriers.

#### Desire for In-Person or On-Demand Help With Telemedicine

Given the complexity of the setup process and the different telemedicine platforms used by different health care providers, participants noted the importance of having in-person help to establish video visits. For example:

I need a person to sit down with me next to my computer to help me set up my account: here’s the icon you click on, the name of your account, where you keep your password, how you enter and use it...I need personal help.

Many recognized that in-person assistance was not always feasible or safe during times of quarantine during the COVID-19 pandemic and hoped for easier access to on-demand assistance with troubleshooting. One participant recalled a story in which she followed all the instructions for downloading the video visit platform and did not understand the step about clearing cookies on her computer and went online to find the information technology desk number for help. She relayed:

until you give thorough instructions, it’s not going to work. A lot of people give up. A live body [to help] would be the best thing.

Another participant emphasized the specialized help needed for older adults:

[My healthcare system] is investing a lot in telemedicine. It would be good to have a team of helpers who could help a patient, mostly older people, and get in touch in advance to help them set up appointments.

Others voiced frustrations with being placed on hold for a long time when trying to call their clinic for assistance.

## Discussion

Of the 249 older adults who completed our survey, most (53%, 132/249) were interested in using telemedicine to connect with care providers through video visits. While older age was associated with decreasing familiarity with technology, it did not diminish interest in telemedicine. Participants identified several barriers regarding telemedicine use, especially in conducting video visits. The top barriers included not knowing how to connect to the platform (including language barriers that make instructions difficult to understand), not being familiar with the technology, difficulty hearing, and lack of interest in seeing providers outside of the clinic.

The digital divide for older adults, who experience challenges with using telemedicine, is well documented [9-12,14,15]. While some have hypothesized as to why these challenges exist from secondary proxies such as insurance data and provider surveys, our study elucidates some of the unique barriers from the perspective of community-dwelling older adults themselves.

We found that older adults are more familiar with telephone than online video platforms, though the majority of participants were interested in learning to use both as a means of connecting with their providers. Similar to a study on older adults’ experiences with technology for socialization [24], we found that, while some older adults are familiar with more widespread technological platforms for social connection, it becomes much more challenging to set up telemedicine platforms for video visits. The challenges of adopting telemedicine for older adults is partly due to a lack of familiarity with video and internet technology and partly due to the challenges of adopting new technological skills in the face of increasing functional deficits such as hearing, vision, memory, and cognition [9]. Given a multitude of institution-specific platforms used for telemedicine, it is important to make sure platforms are streamlined and easy to use or consider adopting platforms that already have widespread social adoption. On demand telephone or in-person support for troubleshooting and caregiver training will also help, as many older adults rely on caregivers and adult children for technology assistance.

Our participants also highlighted the challenges of navigating telemedicine platforms when English is not their first language. Socioeconomic disparities in digital access are well documented [25], and it has been shown that non-White patients, patients who needed interpreter services, and patients who received Medicaid were less likely to have video visits [26]. Actionable steps toward ameliorating these disparities include creating simple instructions in multiple languages for how to use telemedicine platforms and offering language and culturally concordant telemedicine training.

We found that in addition to technology barriers, there are nuanced reasons for reluctance in older adults to conduct video visits. Reluctance to adopt telemedicine may stem from the perception that video visits are inferior to in-person care due to the lack of human touch possible through the physical exam. This is congruent with a national survey of older adults whose chief concern about telemedicine focused on limitations in physical exam and worries about decreased quality of care and connection to providers [27]. However, once older adults have successfully completed a telemedicine visit, they are more willing to continue using telemedicine as part of their care, especially to see providers with whom they have prior established in-person relationships [16]. As telemedicine becomes a greater staple in modern care delivery even beyond the pandemic [28,29], it is important to have clear messaging about the role of telemedicine in augmenting, not replacing, in-person care. When used properly, telemedicine services have the potential to improve health outcomes, access and timeliness of care, and at-home management of chronic disease [30-32]. Improving understanding of telemedicine, specifically increasing education about its role and limitations to the older adult population, may clarify misconceptions and further encourage adoption.

This is a community-based study and has some limitations. Sites A and B are not representative of all older adult residents; we chose these sites to better understand the barriers regarding telemedicine access within our community independent living facilities, as there was a wide range of ages, socioeconomic backgrounds, and primary languages spoken. Furthermore, there may be selection bias given the voluntary nature of the surveys completed; data represent only those who were willing and available to participate.

The COVID-19 pandemic necessitated the rapid scale-up of both provider use and patient adoption of telemedicine. There was little time available to elicit patient perspectives in the process of designing technological platforms for care delivery. Older adults make up many patients in our health care system, though their perspectives are rarely formally elicited. Decreased use of telemedicine exposes this already vulnerable population to further health care inequities. In our study, we surveyed the perspectives of older adults to ascertain their perceived barriers to telemedicine access and highlight themes that further our understanding of the challenges that lead to decreased access to care. Due to site-specific differences in reported telemedicine barriers, any intervention to improve access should be tailored to the specific needs of that site. Our study will not only inform our own quality improvement initiatives in our community but also, we hope, open the door to larger scale studies in understanding the patient experience as telemedicine becomes a larger cornerstone of care delivery.

## Appendix

Multimedia Appendix 1 The survey distributed to older adults residing in the independent living facilities at both sites.

Multimedia Appendix 2 Questions that were asked of older adults that opted for a follow-up interview by phone.

## Abbreviations

IRB: institutional review board
