# Supplementary material for: Barriers to Telemedicine Video Visits for Older Adults in Independent Living Facilities: Mixed Methods Cross-sectional Needs Assessment
Source: JMIR Aging. 2022 Apr 19;5(2):e34326. doi: 10.2196/34326 (PMC9066341; doi:10.2196/34326)
Supplement: Multimedia Appendix 1 [file aging_v5i2e34326_app1.docx]

**Multimedia Appendix 1.** The survey distributed to older adults residing in the independent living facilities at both sites.

Survey for patient and/or caregiver:

1. Are you a (check one)

☐ Resident  ☐ Caregiver answering on behalf of the resident

1. What is your (the resident’s) gender?

☐ Male ☐ Female

1. What is your age?  _______
2. Where do you live?

☐ In Community A

☐ In Community B

☐ In another different living facility: ____________

☐ In my own home by myself

☐ In my own home by with family member(s) or housemates

☐ Other:__________________

1. What is your preferred language?

☐ English    ☐Spanish    ☐Mandarin   ☐Cantonese   ☐Tagalog

☐Vietnamese  ☐Korean  ☐Russian     ☐Other: ____

1. What is your highest level of education

☐ 12^th^ grade or less

☐ Graduated high school or equivalent

☐ Some college, no degree

☐ Associate degree

☐ Bachelor’s degree

☐ Post-graduate degree

1. Where do you get your primary care mainly?

☐ Stanford   ☐Kaiser ☐Sutter Health (PAMF) ☐Other: _________

1. What is your race/ethnicity? Please mark the one box that describes the race/ethnicity category with which you primarily identify.

☐ Hispanic or Latino

☐ White

☐ Black or African American

☐ Asian

☐ Native Hawaiian or Other Pacific Islander:

☐ American Indian or Alaska Native:

☐ Two or More Races

☐ I choose not to self-identify my race/ethnicity at this time.

The next few questions ask about your (the resident’s) experience with devices like a phone, smartphone (iphone or android), or computer.

1. Preferred device for remote visits with your clinician or healthcare team

☐ Regular phone  ☐ Smartphone ☐  iPad/Tablet  ☐ Computer

☐I don’t have any of the above

1. I feel comfortable with smart devices like smartphones, ipads, the computer

☐ Strongly Disagree  ☐ Disagree  ☐  Neither ☐ Agree   ☐ Strongly Agree

1. I know how to get online and use the internet without issues

☐ Strongly Disagree  ☐ Disagree  ☐  Neither ☐ Agree   ☐ Strongly Agree

1. I know how to connect with my healthcare team through video visits

☐ Strongly Disagree  ☐ Disagree  ☐  Neither ☐ Agree   ☐ Strongly Agree

1. I know how to connect with my healthcare team through telephone

☐ Strongly Disagree  ☐ Disagree  ☐  Neither ☐ Agree   ☐ Strongly Agree

The next few questions ask about how you would like to connect with your care team and if you need help with the technology

1. I would like to be able to connect with my healthcare team through video visits

☐ Strongly Disagree  ☐ Disagree  ☐  Neither ☐ Agree   ☐ Strongly Agree

1. I would like to be able to connect with my healthcare team through telephone visits

☐ Strongly Disagree  ☐ Disagree  ☐  Neither ☐ Agree   ☐ Strongly Agree

1. I have someone who can help me access video visits if I have trouble

☐ Strongly Disagree  ☐ Disagree  ☐  Neither ☐ Agree   ☐ Strongly Agree

1. Learning to get better at using technology is worthwhile for me

☐ Strongly Disagree  ☐ Disagree  ☐  Neither ☐ Agree   ☐ Strongly Agree

1. Biggest barriers connecting with my care team through video visits (check up to 3)

☐Difficulty hearing well enough to participate

☐Difficulty seeing well enough to interact with the screen

☐Problems speaking or making oneself understood

☐Problems with attention or memory

☐Not having stable internet connection

☐Not familiar with how to use the technology or internet

☐Not knowing how to get connected to the platform

☐No smart device (ipad,  iphone computer)  available

☐Cannot speak English very well

☐Not interested in seeing provider outside of the clinic

☐No perceived barriers

☐Other: ________________

1. I’m interested in having more training or having someone reach out to help me better connect through video visits with my healthcare team

☐ Strongly Disagree  ☐ Disagree  ☐  Neither ☐ Agree   ☐ Strongly Agree

1. Any other comments?

-----OPTIONAL/DETACH HERE----

Would you be open to having a student call you to help you set-up for a video visit? If yes, please complete the following:

Name:

Best phone number to contact you:

Best times to contact you:
