# Supplementary material for: Barriers to Telemedicine Video Visits for Older Adults in Independent Living Facilities: Mixed Methods Cross-sectional Needs Assessment
Source: JMIR Aging. 2022 Apr 19;5(2):e34326. doi: 10.2196/34326 (PMC9066341; doi:10.2196/34326)
Supplement: Multimedia Appendix 2 [file aging_v5i2e34326_app2.docx]

**Multimedia Appendix 2**. Questions that were asked of older adults that opted for a follow-up interview by phone.

During COVID, how are you getting your medical care?

• Have you seen your healthcare provider since the pandemic started? How did that go?

• Is there anyone you can turn to for help? What resources do you have in your community to help if you need help during COVID?

Have you ever tried to do a video or telephone visit?

• How did it go? (If non English speaking, did your doctor have an interpreter on the line?

What about during in person visits, do they usually have an interpreter?)

• Do you feel like you are reluctant to use technology? If so, why?

• What are the hardest parts of doing video visits or phone visits with your medical team?

• Which part of getting on to the platform did you have trouble with?

• Do you think video visits are as good as in-person visits? What are the biggest limitations?

• Are you more interested in talking to your doctors over the phone or on video if the choice of in person visit is not available?

• What do you think would be helpful in better facilitating video visits?

What do you do to help with memory loss? Hearing loss? Vision issues?

• What resources do you have to help with this?

• Do you have someone you can turn to for help?

• (If you living in an independent living facility with staff): Have the staff in your apartment or any social worker helped you before?
